# Management of Newborns Exposed to Maternal Influenza: A Scoping Review

**DOI:** 10.3390/life16071171

**Published:** 2026-07-15

**Authors:** Agata Kadaj, Paula Trif, Radu Galis, Sophie I. Kramer, Boris W. Kramer, Claudia Maria Jurca, Jan Mazela

**Affiliations:** 1 Department of Neonatology, Poznan University of Medical Sciences, 61-701 Poznan, Poland; 2 Doctoral School of Biological and Biomedical Sciences, University of Oradea, 410073 Oradea, Romania; 3 Department of Neonatology, Emergency County Hospital Bihor, 410167 Oradea, Romania; 4 Department of Medical Sciences, Faculty of Medicine and Pharmacy, University of Oradea, 410073 Oradea, Romania; 5 Department of Neonatology, Technische Universität Dresden, 01307 Dresden, Germany; 6 Department of Preclinical Disciplines, Faculty of Medicine and Pharmacy, University of Oradea, 410073 Oradea, Romania; 7 Regional Center of Medical Genetics Bihor, County Emergency Clinical Hospital Oradea (Part of ERN ITHACA), 410469 Oradea, Romania

**Keywords:** maternal influenza, newborn management, peripartum infection, breastfeeding, rooming-in, infection control, scoping review

## Abstract

**Background:** Maternal influenza during the peripartum period poses a clinically significant risk to newborns, particularly for infants younger than 6 months. This group is especially vulnerable because of immune immaturity and the absence of an approved influenza vaccine for this age group. In light of emerging influenza virus mutations with pandemic potential, current protocols require evaluation with respect to infection prevention. **Objective:** To map and synthesize the available evidence and recommendations on the management of neonates born to mothers with suspected or confirmed influenza, with particular focus on delivery, skin-to-skin contact, breastfeeding, mother–infant separation, and neonatal antiviral therapy. **Sources of Evidence:** We performed a scoping review of the literature and professional guidance documents addressing the perinatal and postnatal management of newborns exposed to maternal influenza, using PubMed, Google Scholar, Web of Science, Embase, WHO, and CDC publications. The available evidence was synthesized narratively across the principal domains of clinical care. **Conclusions:** Current evidence does not support changing the mode of delivery solely because of maternal influenza. When the mother’s clinical condition permits, rooming-in, skin-to-skin contact, and breastfeeding can generally be supported with strict droplet precautions and hand hygiene. These practices provide substantial benefits for bonding, nutrition, and passive immune protection. Temporary separation may be considered in selected cases of severe maternal illness, although consistent evidence of benefit is limited. In neonates with suspected or confirmed influenza, early antiviral treatment should be considered in accordance with current pediatric guidance, whereas routine chemoprophylaxis in infants younger than 3 months remains insufficiently supported by evidence. Overall, recommendations remain heterogeneous, highlighting the need for higher-quality studies and clearer neonatal care pathways.

## 1. Introduction

Influenza affects nearly one billion people worldwide each year. According to the World Health Organization, seasonal influenza causes 3–5 million cases of severe illness annually and is associated with 290,000–650,000 respiratory deaths worldwide [1]. Severe disease is particularly relevant in high-risk groups, including newborns and pregnant or recently postpartum women. Children younger than 5 years and women during pregnancy or within 2 weeks after delivery are more likely to develop severe influenza, particularly in the absence of vaccination [2]. Influenza in pregnancy is associated with substantial maternal and perinatal morbidity and mortality [3,4], while infants younger than 6 months remain at increased risk of severe outcomes and cannot be protected through direct vaccination [5]. In addition, no robust recommendations support routine chemoprophylaxis in infants younger than 3 months [6]. These factors underscore the importance of optimizing intrapartum and postnatal management for newborns exposed to maternal influenza.

After an incubation period of approximately 1–2 days, influenza may present with fever, chills, rhinorrhea, headache, myalgia, malaise, nonproductive cough, sore throat, vomiting, diarrhea, and abdominal pain [7]. Severe cases may be complicated by bacterial lower respiratory tract coinfection, pneumonia, respiratory failure, or croup. Additional reported complications include seizures, myocarditis, pericarditis, acute otitis media, and myositis [8]. Neonates are particularly susceptible because of immune immaturity, including limited T-cell-mediated pathogen recognition and response [9], as well as attenuated antibody responses with reduced early-life IgG production [10]. Higher viral loads have been observed in hospitalized infants than in ambulatory patients [11]. Although many infections are asymptomatic or limited to mild respiratory illness [12], consistent recommendations are needed to protect newborns from potentially severe or fatal disease.

The emergence of a novel influenza virus mutation could precipitate a pandemic, posing a particular threat to mothers in the perinatal period and to their newborns, as indicated by the risk factors outlined above. In this context, we examined the quality and content of existing protocols for perinatal mothers who test positive for influenza and for their newborns, with specific attention to their applicability for a potential future pandemic.

In this scoping review, we summarize literature-based recommendations for delivery management and postnatal care of newborns born to mothers with influenza.

## 2. Materials and Methods

### 2.1. Study Design

We selected a scoping review as the most suitable method to address our research question: “What is the evidence on the management of newborns exposed to maternal influenza?” Our review followed the framework developed by Arksey and O’Malley [13], further refined by Levac et al. (2010) [14]. This approach involves several key steps: identifying the research question, locating relevant studies, selecting studies, charting the data, and finally collating, summarizing, and reporting the findings. The results of our review are presented in accordance with the 2018 PRISMA extension for scoping reviews (PRISMA-ScR) guidelines. The PRISMA-ScR checklist is provided in the Supplementary Materials [15].

### 2.2. Search Strategy

We performed an extensive literature review of relevant articles from the Google Scholar, PubMed, and Web of Science electronic databases. Grey literature and guideline sources were also searched, including WHO, CDC, and UpToDate. The literature search included studies and guidance documents published in English between January 2009 and April 2026, corresponding to the emergence of the 2009 H1N1 influenza pandemic.

The following Boolean logic was used to combine the keywords: (*influenza* OR *flu*) AND (*guideline* OR *prophylaxis* OR *prevention*) AND (*maternal infection* OR *pregnancy*) AND (*newborn* OR *neonatal infection*).

To ensure reproducibility, the complete search strategy was documented for each source. In PubMed, the search string was: (*influenza* OR *flu*) AND (*guideline* OR *prophylaxis* OR *prevention*) AND (*maternal infection* OR *pregnancy*) AND (*newborn* OR *neonatal infection*), limited to English-language publications from January 2009 to April 2026. In Web of Science, the same Boolean structure was applied across topic fields using: TS = ((influenza OR flu) AND (guideline OR prophylaxis OR prevention) AND (“maternal infection” OR pregnancy) AND (newborn OR “neonatal infection”)), with the same date and language limits. In Google Scholar, the search was adapted to the platform using the phrase: influenza OR flu guideline prophylaxis prevention “maternal infection” pregnancy newborn “neonatal infection”, screened within the same publication period.

Guideline and grey-literature searches were conducted on the WHO and CDC websites using combinations of the same terms, particularly *influenza*, *pregnancy*, *newborn*, *prophylaxis*, and *prevention*. UpToDate was searched using the terms *influenza pregnancy newborn*, *maternal influenza neonatal management*, and *influenza prophylaxis neonate*. Search results were screened for relevance to maternal influenza, neonatal exposure, infection prevention, breastfeeding, mother–infant contact, separation policies, and neonatal antiviral treatment or prophylaxis.

### 2.3. Eligibility for Research Question

We addressed the question “What is the evidence on the management of newborns exposed to maternal influenza?”

We applied the Population, Concept, and Context (PCC) framework to define the eligibility of our research question as shown in Table 1.

### 2.4. Eligibility Criteria and Study Selection

#### 2.4.1. Inclusion Criteria

Studies were included if they met the following criteria:

- Addressed the management of neonates born to mothers with confirmed influenza infection during the pregnancy.
- Focused on at least one relevant domain of neonatal or perinatal care, of which:
  ○ Mode of delivery,
  ○ Rooming-in,
  ○ Skin-to-skin contact,
  ○ Mother–infant separation,
  ○ Breastfeeding,
  ○ Antiviral treatment or prophylaxis.
- Included neonates/newborns in the first 28 days of life exposed to maternal influenza.
- Were original research articles, review articles, consensus statements, clinical guidelines, or recommendations issued by professional organizations.
- Were published in peer-reviewed journals or as official guidance documents.
- Were available in English.

#### 2.4.2. Exclusion Criteria

Exclusion criteria included:

- Studies focused exclusively on influenza during the pregnancy but without neonatal management data,
- Animal studies,
- Studies involving older infants or pediatric populations without separate neonatal data,
- Studies without full-text access.

The study selection was carried out in three stages. Initially, one reviewer (A.K.) screened the titles from the databases based on the eligibility criteria. An Endnote library was created using Endnote 20(Clarivate, Philadelphia, PA, USA), and all articles deemed eligible after title screening were exported into this library. Next, two reviewers (A.K. and P.T.) reviewed the abstracts and full articles. Any disagreements at the abstract screening stage were resolved through discussion until consensus was reached. Discrepancies during full article screening were addressed by involving a third reviewer (R.G.).

### 2.5. Charting of Data

Data were extracted using a standardized charting form developed in accordance with PRISMA-ScR recommendations. Extracted variables included author and year of publication, study characteristics and aim, population details, maternal influenza status, and outcomes reported, as seen in Table 2.

## 3. Results

### 3.1. Screening Results

**Figure 1:**
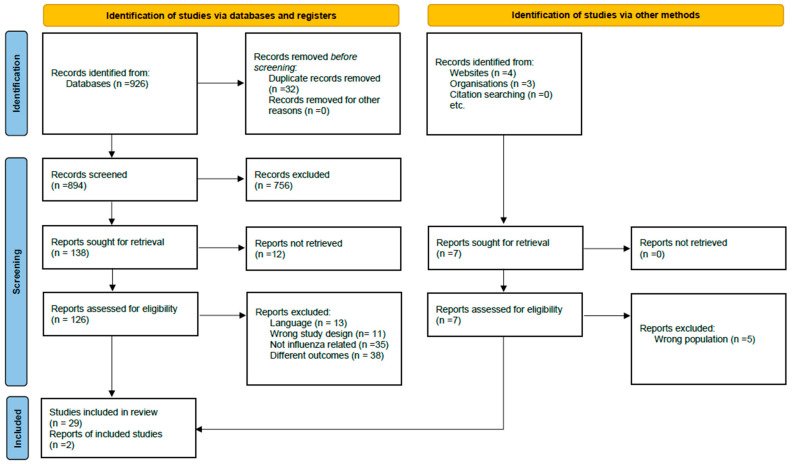
PRISMA flow diagram.

Our search identified 933 potentially eligible articles. After title screening, articles were eligible for abstract screening. After removing 32 duplicates, 901 articles proceeded to abstract screening. A total of 756 articles were excluded after abstract screening, leaving 145 for full-article screening; 31 articles were included in the data extraction. Figure 1 shows the flow diagram of the screening results.

### 3.2. Characteristics of Included Studies

Characteristics of included articles are presented in Table 2.

### 3.3. Study Findings

The main themes identified through thematic analysis of the included studies were delivery, skin-to-skin contact, breastfeeding, mother–infant separation, maternal vaccination, and neonatal antiviral treatment. The quantity and quality of the available evidence varied considerably across these management strategies. To improve transparency, Table 3 summarizes the strength of the available evidence supporting each intervention. The findings presented below are interpreted in the context of these differences in evidence.

#### 3.3.1. Delivery

The available evidence supporting delivery management consists primarily of professional guidelines, observational studies, and systematic reviews, which consistently indicate that maternal influenza alone should not determine the mode of delivery [17]. Delivery decisions should instead be based on maternal and fetal indications [40]. Maternal influenza may still adversely affect neonatal outcomes through its association with preterm birth, fetal growth restriction, and increased perinatal morbidity, which may in turn contribute to a higher cesarean delivery rate [22]. When cesarean delivery is performed without clear maternal or fetal justification, it may increase maternal surgical risk and contribute to prolonged mother–infant separation, delayed breastfeeding initiation, reduced colostrum exposure, and increased formula feeding.

Separation for maternal reasons may also complicate neonatal adaptation and increase infection-related morbidity in hospitalized newborns [44,45]. There is no evidence to support omitting essential interventions in the first minutes of life because of maternal influenza, including delayed cord clamping, drying and thermal protection, and stimulation of breathing when indicated.

A key clinical question is whether skin-to-skin contact increases the risk of neonatal influenza transmission. Available evidence does not support routinely withholding skin-to-skin care solely because of maternal influenza, particularly when the mother is clinically stable and appropriate infection-control measures are in place. Early skin-to-skin contact may support physiologic adaptation, promote bonding, and facilitate breastfeeding, which may provide passive immunologic benefit. Any potential transmission risk should therefore be balanced against these benefits, with decisions individualized according to maternal condition, neonatal vulnerability, and the feasibility of droplet precautions and meticulous hand hygiene.

Skin-to-skin contact may also support exclusive breastfeeding, thereby enhancing passive immune protection and potentially reducing the newborn’s exposure to additional caregivers, staff, and unnecessary procedures [45]. When provided, appropriate precautions should be maintained, including maternal face-mask use and careful hand hygiene, especially after contact with respiratory secretions [16]. These measures are central to reducing transmission risk while preserving mother–infant proximity, bonding, and breastfeeding.

#### 3.3.2. Breastfeeding

Human milk contains antiviral components, including lactoferrin, human milk oligosaccharides, and milk fat globule membranes, that may help protect infants against influenza by interfering with viral binding, entry, and replication. Although transmission of influenza virus through human milk appears to be rare, infected mothers may transfer protective antibodies to their infants [24]. Breastfeeding with enhanced precautions therefore remains the preferred and generally safe approach to neonatal nutrition while supporting passive immune protection [37]. Measures such as meticulous hand hygiene, mask use during breastfeeding, cough etiquette, and, when feasible, maintaining distance between the infant’s crib and the mother’s bed are reasonable strategies to reduce transmission risk.

Influenza vaccination during pregnancy benefits both mothers and infants [26]. Maternal vaccination reduces laboratory-confirmed influenza and influenza-related hospitalization in infants, in part through the type and timing of antibodies appearing in maternal blood and human milk. Natural infection appears to stimulate predominantly influenza-specific IgA responses, whereas vaccination induces a more IgG-dominant response [25]. This combination of mucosal immunity, including secretory IgA and secreted IgM, together with serum IgG, may reduce infection risk and partially compensate for neonatal immune immaturity [35], and no influenza vaccine is licensed for this age group, further underscoring the importance of maternal vaccination during pregnancy [32]. A 2020 meta-analysis found that maternal immune responses were associated with a 34% reduction in infant influenza risk or attenuation of disease severity [26,32]. Additional benefits of maternal immunization include reductions in preterm birth, small-for-gestational-age birth, and stillbirth [30]. Highest cord-blood antibody titers have been observed when vaccination occurred at least 4 weeks before delivery [43]. Because influenza-specific IgG appears in peripheral blood approximately 7 days after infection onset and around 14 days after vaccination, a newborn delivered within 7 days after maternal influenza diagnosis, particularly in the absence of prior maternal vaccination, is likely to receive limited passive protection through the placenta and possibly through breast milk [25,28,42]. Although direct influenza data are limited, studies of other aerosol-transmitted viruses such as SARS-CoV-2 suggest that IgG and IgA in human milk may begin to rise about 10 days after symptom onset and persist for several months [25,31]. Exclusive breastfeeding by vaccinated mothers may therefore represent the best available strategy for supporting protection against respiratory illness during the first 6 months of life and may also encourage breastfeeding continuation [25,31,42]. Pregnant women and women in the early postpartum period with influenza should receive antiviral therapy such as oseltamivir to reduce maternal morbidity and mortality [37,39]. Oseltamivir inhibits viral neuraminidase, reducing the release of new virions from infected host cells and attenuating disease severity [20]. Treatment shortens illness duration by approximately 29 h [27], and can be used during breastfeeding [30]. Analyses of pregnant women exposed to oseltamivir in different countries have not shown evidence of adverse fetal effects or increased congenital anomalies [21,41]. Nevertheless, because oseltamivir is not formally recommended during pregnancy in some summaries of product characteristics, its use in pregnant and lactating women remains largely off-label [23,37].

While breastfeeding provides important postnatal immunologic protection, maternal vaccination during pregnancy complements these benefits by establishing passive immunity before birth.

Maternal influenza vaccination emerged as the most effective preventive strategy. Vaccination during pregnancy not only reduces the risk of influenza-related illness and hospitalization in pregnant individuals, but also provides passive immune protection to the newborn through transplacental antibody transfer. This protection is particularly important during the first months of life, when infants are at increased risk of severe influenza but are still too young to receive influenza vaccination themselves. Recent evidence supports this preventive effect. A New Vaccine Surveillance Network study of infants younger than 6 months reported maternal vaccine effectiveness of 34% against medically attended influenza, 39% against influenza-associated hospitalization, and 53% among infants younger than 3 months [46]. Updated data from the same surveillance network across the 2016–2017 through 2024–2025 influenza seasons found similar overall effectiveness of 34% and 42% effectiveness against hospitalization [47]. A large Kaiser Permanente cohort including more than 245,000 infants found that maternal vaccination was associated with a 44.4% reduction in infant influenza during the first 6 months of life, with greater protection when vaccination occurred in the second or third trimester [48]. In Europe, a population-based cohort study from Lombardy, Italy, reported estimated maternal influenza vaccine effectiveness of 69.7% against influenza-related hospital or emergency department care in infants younger than 6 months [48]. These recent findings are consistent with earlier pooled evidence, including a systematic review and meta-analysis that found a 48% reduction in laboratory-confirmed influenza infection among infants younger than 6 months after maternal vaccination [49]. In pregnant individuals, seasonal influenza vaccination has also been associated with reduced influenza-related acute care use; for example, a 2023–2024 multicentre study estimated 46% effectiveness against influenza-associated emergency department or urgent care encounters among pregnant women [50]. Compared with post-exposure measures such as antiviral prophylaxis, isolation, or temporary mother–infant separation, vaccination offers a proactive and broadly applicable approach that can prevent infection before neonatal exposure occurs. Therefore, timely influenza vaccination during pregnancy should be considered a central component of perinatal influenza prevention, alongside hygiene measures, droplet precautions, and appropriate clinical management when maternal infection is suspected or confirmed.

#### 3.3.3. Mother–Infant Separation

Among the interventions evaluated in this review, mother–infant separation is supported by the weakest evidence base, with current recommendations relying largely on expert consensus, infection-control guidance, and indirect evidence rather than comparative clinical studies.

There is little to no randomized clinical trial data to guide this difficult decision. Each case should be assessed individually, taking into account local guidance and hospital capacity [18]. If the mother’s condition allows, a term infant should generally remain with the mother. In cases of severe maternal illness or respiratory failure, temporary care by healthcare staff may be necessary until maternal status improves. When mother and infant remain together, they should ideally be cared for in a separate room arranged to reduce the infant’s exposure to respiratory aerosols [34]. A distance of 2 m from the mother’s bed has been proposed as a precautionary measure based on aerosol and droplet transmission theory, although direct supporting evidence remains limited. Visitor access should be restricted, ideally to one key family member [33]. Face-mask use during breastfeeding and close contact, careful hand hygiene, and physical distancing when direct care is not being provided are reasonable measures to reduce transmission risk [18]. Management may differ for preterm infants, who are at greater risk of infection-related morbidity. When prematurity necessitates neonatal intensive care admission, maternal contact and visitation may need to be restricted for at least 5–7 days after illness onset to reduce transmission risk to the infant, other patients, and healthcare staff [17,18]. Since the H1N1 pandemic, relatively few studies have evaluated these recommendations or generated new evidence-based practice principles [51]. Current CDC guidance considers temporary separation of a clinically ill mother from her newborn as one option to reduce influenza transmission [18]. The duration of separation should depend on maternal clinical status. Reunification may be considered once the mother has been afebrile for at least 24 h without antipyretics and is able to control respiratory secretions and cough. If separation is not feasible or not pursued, strict droplet precautions should be maintained, including face-mask use during contact with the infant, hand hygiene before and after handling the newborn, and keeping the infant’s bassinet approximately 2 m from the mother’s bed when possible. Face-mask use and cough control should continue for 7 days after illness onset or until 24 h after resolution of fever and respiratory symptoms, whichever is longer [18].

#### 3.3.4. Treatment of the Infant

Infants with confirmed influenza A or B by nasal swab, or with suspected influenza, should receive first-line antiviral therapy as early as possible, ideally within 48 h of symptom onset [9]. Because newborns are at increased risk of severe disease, antiviral treatment is recommended for infected neonates. According to American Academy of Pediatrics recommendations for 2024–2025, oseltamivir dosing in newborns should be based on postmenstrual age [6]. For infants older than 40 weeks’ postmenstrual age, the recommended dose is 3 mg/kg per dose twice daily for 5 days; for those 38–40 weeks’ postmenstrual age, 1.5 mg/kg per dose twice daily for 5 days; and for those 28–38 weeks’ postmenstrual age, 1 mg/kg per dose twice daily for 5 days [6]. Chemoprophylaxis is recommended for selected children older than 3 months who are at increased risk of severe influenza [6]. In infants younger than 3 months, the main limitation is not known toxicity but the lack of sufficiently robust evidence to support effectiveness [38]. Even if chemoprophylaxis may offer potential benefit in infants deprived of passive immunity, it should not be considered a substitute for maternal vaccination. The most commonly reported adverse effects of oseltamivir include nausea, vomiting, abdominal pain, diarrhea, headache, insomnia, and vertigo [36]. Hepatotoxicity, reflected by elevated aminotransferase levels, has been reported in approximately 2% of treated patients [29]. Neurologic events, including seizures and mood changes, have been described in approximately 0.5% of the studied population, although these symptoms may be difficult to distinguish from manifestations of influenza itself [19]. More serious adverse events are rare, occurring in fewer than 1% of patients, and include arrhythmia, gastrointestinal bleeding, erythema multiforme, Stevens–Johnson syndrome, toxic epidermal necrolysis, confusion, seizures, and other neuropsychiatric events [36]. Given the low incidence of adverse effects, oseltamivir is generally considered safe for infants and children [27].

Chemoprophylaxis as a treatment may be considered since a vaccination as proper prevention is currently not licensed in infants younger than 3 months.

## 4. Discussion

This review summarizes the available evidence on the management of neonates born to mothers with suspected or confirmed influenza. Across the included studies, four closely related themes emerged: mode of delivery, breastfeeding, mother–infant separation, and neonatal antiviral treatment. Taken together, the findings suggest that care should not be driven by infection prevention alone, but by a balanced assessment of transmission risk, maternal clinical status, neonatal vulnerability, and the well-established benefits of early mother–infant contact and breastfeeding.

A central finding is that maternal influenza alone does not appear to justify a change in delivery mode or the omission of routine immediate newborn care. Delivery decisions should therefore remain based on obstetric and fetal indications rather than maternal infection status alone. This is clinically important because unnecessary cesarean delivery may increase maternal morbidity and can indirectly affect neonatal outcomes by delaying skin-to-skin contact, breastfeeding initiation, and early bonding. The evidence reviewed supports an individualized approach in which standard perinatal practices are preserved whenever maternal and neonatal conditions allow, while droplet precautions and hand hygiene are used to reduce exposure risk.

Mother–infant separation remains the most complex and ethically sensitive issue identified in this review. While temporary separation may reduce exposure in selected situations, especially when the mother is severely ill or the infant is preterm or otherwise vulnerable, routine separation may also disrupt breastfeeding, bonding, and maternal psychological well-being. The reviewed literature therefore supports a nuanced risk-based strategy rather than a universal policy. For clinically stable mothers and term infants, rooming-in with precautions may be appropriate. In contrast, temporary separation may be justified when maternal illness is severe, when infection-control measures cannot be reliably maintained, or when neonatal vulnerability substantially increases the expected harm of infection. The COVID-19 pandemic has yielded data regarding “skin-to-skin contact” and “rooming-in”. However, high-quality studies—such as randomized controlled trials (RCTs)—focusing on “maternal-infant separation” are non-existent. The overall importance of skin-to skin contact prevents further studies on separation, since there is no longer any equipoise. The lack of studies is therefore in part ethically motivated. We do not perceive any short term solution to the lack of equipoise which results in a disparity of data quality and volume on the different approaches of reducing the risk of transmission by distance or separation.

The treatment findings reinforce the importance of early recognition and prompt antiviral therapy in neonates with suspected or confirmed influenza. Because newborns have immature immune responses and are at increased risk of severe disease, delays in treatment may have important clinical consequences. Oseltamivir remains the main therapeutic option discussed in current guidance, with dosing adjusted according to postmenstrual age. However, the evidence base for chemoprophylaxis in infants younger than 3 months remains limited. This distinction is important: treatment of suspected or confirmed infection is supported more strongly than prophylactic use, and prophylaxis should not be considered a replacement for maternal vaccination, breastfeeding support, and infection-control measures.

Maternal influenza vaccination emerges as a key preventive strategy across several themes. Vaccination during pregnancy can reduce maternal disease burden and provide passive protection to the infant during the first months of life, when direct vaccination is not available. The timing of vaccination is likely to be relevant, as antibody transfer may be limited when infection occurs shortly before delivery or when vaccination has not preceded birth by enough time to generate an adequate immune response. The vaccination is therefore the preferred option due to the lack of research to determine whether prophylaxis in selected high-risk infants may offer net benefit rather than neonatal chemoprophylaxis. These findings highlight the need to integrate influenza vaccination counselling into routine antenatal care and to ensure that postpartum and lactation-related questions are addressed clearly.

Available evidence suggests that guidance for the management of newborns born to mothers with influenza remains limited and, in some areas, inconsistent. Prevention of neonatal influenza relies primarily on maternal vaccination rather than neonatal chemoprophylaxis, although further research is needed to determine whether prophylaxis in selected high-risk infants may offer net benefit. The commonly cited distance of 6 feet (2 m) has long been considered sufficient to reduce transmission risk, yet more recent work suggests that this assumption may be overly simplistic [33,34,52]. Depending on airflow, ventilation, and environmental conditions, respiratory particles may travel beyond 2 m. Experimental and modeling studies suggest that droplets expelled during coughing and sneezing may spread farther than 2 m, whereas particles generated during quiet breathing generally remain within a shorter range [53]. Thus, a 2 m distance alone may be insufficient and should not be considered a definitive infection-control measure in the absence of additional precautions. COVID-19 has been studied more extensively than influenza because of the recent pandemic. Although both infections are transmitted primarily through respiratory droplets, findings from COVID-19 research cannot always be directly applied to influenza, particularly in neonatal settings. The following criteria are similar in COVID-19 and influenza in a hospital setting thus parallels can be drawn. Like COVID-19, neonatal influenza is most often acquired after birth through horizontal transmission. Common sources include infected family members, visitors, or hospital staff. Vertical transmission during pregnancy is considered much less common. Symptom-based screening for visitors to neonatal intensive care units (NICUs) is consistent with prevention approaches used for COVID-19 and neonatal pneumonia. Hospitals can further reduce transmission risk by combining screening with standard infection-control practices. This comprises enforced Standard and Droplet Precautions, including strict hand hygiene before and after patient contact. The universal use of masking and appropriate personal protective equipment (PPE) for healthcare workers and ill household contacts. In addition, a “cocooning” approach by ensuring that all close caregivers are fully vaccinated, should be applied to lower the risk of community-acquired influenza reaching the newborn or mother. This comparison is thus limited to shared transmission mechanisms or operational workflows to fill the current gaps in neonatal influenza guidance.

## 5. Limitations

This scoping review has several limitations. This review focused primarily on strategies for preventing neonatal influenza transmission. Consequently, broader aspects of neonatal care, including breastfeeding success, maternal–infant bonding, neonatal stress, and maternal mental health, were not examined in depth. These outcomes are important when evaluating practices such as mother–infant separation and should be addressed in future studies, particularly in the context of preparedness for future influenza pandemics. The included evidence was heterogeneous with respect to study design, populations, interventions, and reported outcomes. The inclusion of guidelines and grey literature introduced variability in methodological rigor, while some recommendations were based on limited primary evidence. Only English-language publications were included, which may have resulted in language bias. Additionally, as a scoping review, this study aimed to map the existing literature rather than critically appraise study quality or conduct meta-analysis since it fell outside the defined objectives of the review, which aligns directly with standard JBI methodology.

## 6. Conclusions and Knowledge Gaps

Further research is needed to clarify the importance of skin-to-skin contact and rooming-in versus temporary maternal–newborn separation in preventing neonatal influenza transmission while preserving maternal bonding and breastfeeding. The lack of evidence in clinical studies for separation is also caused by the apparent lack of equipoise. In addition, infants younger than 3 months are at increased risk of influenza-related morbidity and mortality. The role of post-exposure prophylaxis in this population for whom no licensed vaccine is currently available needs further study [6]. However, evidence remains insufficient to determine the efficacy, safety, and optimal indications for chemoprophylaxis in this age group. Individual decisions of physicians and the clinical care groups are needed in the current situation.

## Figures and Tables

**Table 1:** Framework for determining the eligibility of the research question.

PCC Element	Description
Population	Neonates/newborns exposed to maternal influenza infection
Concept	Perinatal and postnatal management strategies and outcomes
Context	Delivery room and neonatal unit management, or postpartum care settings

**Table 2:** Data extraction table for studies and guidance documents included in the scoping review.

Author	Year of Publication	Objective of the Study	Study Design/Document Type	Study Population/Target Population	Maternal Influenza Status	Outcomes Reported
Baek [16]	2014	To prevent and control seasonal influenza	Practice guideline	General population	/	Antiviral therapy; prophylaxis
Cantey [17]	2013	To describe the experience of promoting rooming-in and breastfeeding during maternal influenza infection	Prospective study	Maternal and neonatal	Confirmed	Delivery mode; breastfeeding; mother–infant separation; neonatal outcomes; prophylaxis
Committee on Infectious Diseases [6]	2025	To provide recommendations on prophylaxis and control of influenza in children	Practice guideline	Infants	/	Antiviral therapy; prophylaxis
CDC * [18]	2020	To provide recommendations for the prevention and control of influenza in the peri- and postpartum settings	Policy statement	Maternal and neonatal	/	Prophylaxis; mother–infant separation
Dalvi [19]	2011	To emphasize adverse reactions of oseltamivir in children	Prospective study	Infants	/	Antiviral therapy
Davies [20]	2010	To present the pharmacokinetics of oseltamivir	Narrative review	General population and infants	/	Antiviral therapy
Ehrenstein [21]	2018	To report birth defects after oseltamivir use during pregnancy	Observational study	Maternal and neonatal	/	Antiviral therapy
Fell [22]	2017	To report birth outcomes after maternal influenza	Systematic review	Neonatal	/	Neonatal outcomes
Fodor [23]	2024	To present the pharmacokinetics of oseltamivir in lactating influenza mothers	Observational study	Maternal	Confirmed	Antiviral therapy
Francese [24]	2023	To provide information about viral pathogens and human milk	Narrative review	Maternal	/	Breastfeeding; antiviral therapy
Hunagund [25]	2022	To summarize the immunologic benefits of human milk after viral infections	Narrative review	Maternal and neonatal	/	Breastfeeding; prophylaxis
Jarvis [26]	2020	To report the effectiveness of influenza vaccination on child health outcomes	Meta-analysis	Maternal and neonatal	/	Prophylaxis; breastfeeding; neonatal outcomes
Jefferson [27]	2014	To assess the efficacy and safety of oseltamivir in children and adults	Systematic review	General population and neonatal	/	Antiviral therapy
Kumar [7]	2017	To present the evolution and complications of influenza	Narrative review	Neonatal	/	Neonatal outcomes
Krammer [28]	2019	To review the differences between natural infection and vaccination in terms of the antibody responses	Narrative review	General population	/	Prophylaxis
Malosh [29]	2018	To assess the efficacy and safety of oseltamivir in children	Meta-analysis	Infants	/	Antiviral therapy
Omer [30]	2011	To evaluate the association between receipt of inactivated influenza vaccine during pregnancy and prematurity and small for gestational age	Retrospective cohort study	Maternal and neonatal	/	Antiviral therapy; breastfeeding; neonatal outcomes
Perl [31]	2021	To evaluate antibody transfer from mother to child	Prospective cohort study	Maternal and neonatal	/	Prophylaxis; breastfeeding
Rand [32]	2023	To emphasize the importance of maternal vaccination	Narrative review	Maternal	/	Prophylaxis; neonatal outcomes
Randall [33]	2021	To present the transmission of respiratory infectious diseases	Narrative review	/	/	Mother–infant separation
Sarhan [34]	2021	To examine the feasibility of social distancing	Computational modeling study	/	/	Mother–infant separation
Steinhoff [35]	2010	To present antibody data for mothers and infants after influenza vaccine during pregnancy	Randomized control study	Maternal and neonatal	/	Prophylaxis
Sur [36]	2026	To identify adverse outcomes of oseltamivir use	Narrative review	Maternal and neonatal	/	Antiviral therapy
Tanaka [37]	2009	To summarize information about the safety of neuraminidase inhibitors for treatment of influenza in pregnant and breastfeeding women	Literature review	Maternal and neonatal	/	Antiviral therapy; breastfeeding
UpToDate [38]	2026	To provide recommendations on the use of antiviral medications for the prevention of seasonal influenza in children	Narrative review	Infants	/	Antiviral therapy
Van Bennekom [39]	2019	To investigate associations between oseltamivir and specific birth defects	Retrospective study	Maternal	/	Antiviral therapy
Wielgos [40]	2018	To summarize recommendations for caesarian sections	Practice guideline	Maternal	/	Delivery mode
Wolf [9]	2023	Evidence-based guideline on prevention, diagnosis and management of influenza in children	Narrative review	Infants	/	Antiviral therapy
Wollenhaupt [41]	2014	To reassess the safety of oseltamivir during pregnancy	Review	Maternal	/	Antiviral therapy
Yang [42]	2023	To determine antibody titers in human milk after influenza vaccination	Prospective study	Maternal	/	Prophylaxis; breastfeeding
Zhang [43]	2026	To examine birth outcomes after influenza or vaccination	Retrospective cohort study	Maternal and neonatal	Confirmed or suspected	Prophylaxis

* CDC: Centers for Disease Control and Prevention.

**Table 3:** Strength of the available evidence supporting the identified interventions. The categories “High”, “Moderate”, and “Limited” reflect the overall strength of the evidence identified in this scoping review and are intended to summarize the available literature rather than represent a formal GRADE assessment.

Intervention/Management Strategy	Main Evidence Base Identified in This Review	Strength of Evidence	Interpretation
Delivery	Guidelines, observational studies, systematic reviews	Moderate-quality	Maternal influenza alone should not determine the mode of delivery; decisions should remain based on obstetric and fetal indications.
Skin-to-skin contact/kangaroo care	Multiple randomized and observational studies	High-quality	Should generally be supported when the mother is clinically stable and infection-control precautions can be maintained.
Rooming-in	Prospective clinical data, guideline recommendations, and indirect evidence from COVID-19 literature	Moderate- to high-quality	Supported for clinically stable mothers and term infants with droplet precautions and hand hygiene.
Mother–infant separation	Expert opinion, infection-control guidance, and indirect evidence; no randomized clinical trials	Limited	When maternal illness is severe or infection-control measures cannot be maintained, temporary separation may be considered following individualized clinical assessment.
Breastfeeding	Reviews, observational studies, immunologic evidence, and guideline recommendations	Moderate- to high-quality	Breastfeeding is generally supported; influenza transmission through milk appears rare and passive immune protection is biologically plausible.
Maternal influenza vaccination	Randomized trial, cohort studies, systematic review/meta-analysis, and prospective immunologic studies	High-quality	Strongest preventive intervention for reducing maternal influenza and providing passive infant protection.
Neonatal antiviral treatment	Pediatric guidance, systematic reviews/meta-analyses, pharmacokinetic studies, and safety data	Moderate-quality	Oseltamivir treatment is supported for suspected or confirmed neonatal influenza, with dosing according to postmenstrual age.

## Data Availability

No new data were created or analyzed in this study. Data sharing is not applicable to this article.

## Supplementary Materials

The following supporting information can be downloaded at: https://www.mdpi.com/article/10.3390/life16071171/s1, Preferred Reporting Items for Systematic reviews and Meta-Analyses extension for Scoping Reviews (PRISMA-ScR) Checklist [15].
